# Surgical Experiences in South Africa, 1899-1900

**Published:** 1902-03

**Authors:** 


**Surgical Experiences in South Africa, 1899-1900.** By George Henry Makins. Pp. xvi., 493. London: Smith, Elder & Co. 1901.

These surgical experiences form a most comprehensive account of certain injuries received in battle; but this work, most excellent as it is as far as it goes, must be supplemented by a treatise which deals more fully with the injuries caused by shells, before a complete knowledge of the latest military surgery is gained. The details of shell surgery (if we may use such a term) will be best studied by perusing such authors as have described the surgery in connection with the Natal Army.

The author must have studied the individual cases of several thousand injuries, and having been attached to all three varieties of hospitals—namely, Field, Stationary, and Base,—he has had the opportunity of doing a vast amount of work, and he has placed the work done in a clear and comprehensive manner before the medical profession at home.

We are glad to see the author favours the Indian type of Field Hospital when comparing it with the Home type; we fully endorse this view, although we understand this type of Field Hospital is not generally preferred by the "Home Authorities."

We are pleased to read the very sound remarks concerning female nurses, and we feel certain that after the favourable opinions that have now been so often expressed, that the authorities at head-quarters will in future make such large additions to the nursing staff of the Army as will ensure the the sick and wounded soldier being chiefly nursed by women.

The author has perhaps been altogether too mild in his criticisms dealing with those persons (many of whom were totally unacquainted with hospital requirements) who had not a single good word to say for the hospital management of this war; and we are gratified to see that such a well-informed civilian, and one who has done so much magnificent work during nine months in the South African hospitals, is able to give us such reassuring testimony of the good work done in our military and other hospitals during the Boer War.

It is impossible for us to discuss the various chapters in detail; we advise all surgeons to read carefully their contents, which are finely illustrated with numerous instructive drawings and skiagrams. We consider this work is one of the best of the recent additions to military surgery.

